# Anti-Fas IgM monoclonal antibody (anti-Fas mAb) effect on haemophilic arthropathy (HA) synoviocytes

**DOI:** 10.1186/ar3566

**Published:** 2012-02-09

**Authors:** Serena Guiducci, Eloisa Romano, Claudia Ceccarelli, Daniela Melchiorre, Mirko Manetti, Anna F Milia, Lidia Ibba Manneshi, Kusuki Nishioka, Marco Matucci Cerinic

**Affiliations:** 1 Dept Medicine, Division of Rheumatology, University of Florence, Florence, Italy; 2 Dept Anatomy, Histology and Forensic Medicine, University of Florence, Florence, Italy; 3 Dept Rheumatology, Institute of Medical Science, Tokyo Medical University, Tokyo, Japan

## Background

Haemophilic arthropathy (HA), which shares some clinical and biological injury characteristics with rheumatoid arthritis (RA), is characterized by chronic proliferative synovitis and cartilage destruction. Anti-Fas mAb specifically targets the Fas molecule, which is expressed and activated on the cell surface of inflammatory synovial cells and plays a key role for induction of apoptosis. Caspases are the final executioners of apoptosis and their activation requires proteolytic processing of inactive zymogen into activated fragments.

### Aim

To evaluate the effects of anti-Fas mAb on HA synoviocytes and its capacity of inducing apoptosis analysing caspase 3 activity.

## Methods

HA synoviocytes were incubated with IgM 1000 ng/ml (control), TNFalpha 10 ng/ml, FGF 10 ng/ml, CH11 100 ng/ml (positive control of apoptosis) with or without anti-Fas mAb at different concentrations (from 0,1 to 1000 ng/ml) for 24 h. RA and healthy synoviocytes were used as controls. To measure cell proliferation/citotoxicity, the WST-1 assay has been performed. Caspase 3 activity has been evaluated with ELISA kit and western blot.

## Results

Anti-Fas mAb induced a citotoxic effect in HA (p < 0,001 for any dose), healthy (p < 0,001 at 100 and 1000 ng/ml) and RA synoviocytes (p < 0,05 for any dose) reaching a maximum effect at 1000 ng/ml. After stimulation with anti-Fas mAb combined with TNFalpha, there was a citotoxic effect on healthy (p < 0,001 at 10, 100, 1000 ng/ml anti-Fas mAb), RA (p < 0,001 for any dose) and HA synoviocytes (p < 0,005 at 1, 10, 100 and 1000 ng/ml anti-Fas mAb). After stimulation with anti-Fas mAb combined with FGF, there was a citotoxic effect on healthy, RA and HA synoviocytes (p < 0,001 for any dose). Caspase 3 levels were increased in HA synoviocytes after anti-Fas mAb treatment in a dose-dependent manner, even after co-stimulation with TNFalpha (p < 0,001 for any stimulus). CH11 induced an increase of caspase 3 levels in HA synoviocytes more than RA synoviocytes. Western blot showed that HA synoviocytes had higher levels of activated caspase 3 compared to RA synoviocytes after stimulation with Anti-Fas mAb, CH11 and co-stimulation with TNFalpha.

## Conclusion

Anti-Fas mAb has a dose-dependent citotoxic effect on HA synoviocytes, even when associated with TNFalpha and FGF. Anti-Fas mAb is effective in increasing caspase 3 levels in HA synoviocytes in a dose-dependent manner. HA synoviocytes show higher levels of activated caspase 3 compared to RA synoviocytes. Our results suggest that anti-Fas IgM mAb may favour the induction of apoptosis in HA synoviocytes.

